# Left Ventricular Stiffness in Adolescents and Young Adults with Repaired Tetralogy of Fallot

**DOI:** 10.1038/s41598-017-01448-2

**Published:** 2017-04-28

**Authors:** Clement Kwong-man Yu, Wilfred Hing-sang Wong, Vivian Wing-yi Li, Yiu-fai Cheung

**Affiliations:** Department of Paediatrics and Adolescent Medicine, LKS Faculty of Medicine, The University of Hong Kong, Hong Kong, China

## Abstract

Left ventricular (LV) remodeling after tetralogy of Fallot (TOF) repair may influence LV stiffness. We hypothesized that LV stiffness is altered after TOF repair and related to myocardial calibrated integrated backscatter (cIB) and LV diastolic myocardial deformation. Seventy-seven TOF patients and 80 controls were studied. LV stiffness was assessed by diastolic wall strain (DWS) as defined by (LVPW_systole_-LVPW_diastole_)/LVPW_systole_, where LVPW is LV posterior wall thickness, and stiffness index as defined by (E/*e*/LV end-diastolic dimension), where E and *e* are respectively early diastolic transmitral inflow and mitral annular velocities. Septal and LVPW cIB and LV diastolic strain rates were determined. Patients had significantly lower DWS (p < 0.001), higher stiffness index (p < 0.001), and greater cIB (p < 0.001). LV DWS correlated negatively with LV stiffness index (r = −0.31, p < 0.001), septal cIB (r = −0.21, p = 0.01), E/*e* ratio (r = −0.30, p < 0.001) and RV end-diastolic area (r = −0.31, p < 0.001), and positively with LV early (r = 0.33, p < 0.001) and late (r = 0.20, p = 0.01) diastolic strain rates and RV fractional area change (FAC) (r = 0.24, p = 0.003). Multivariate analysis revealed E/*e* (β = −0.26, p = 0.008), RV end-diastolic area (β = −0.20, p = 0.02), and RV FAC (β = 0.18, p = 0.01) as significant correlates of DWS. Left ventricular stiffening occurs after TOF repair and is related to impaired LV diastolic myocardial deformation, myocardial cIB, and RV volume overload.

## Introduction

Right ventricular (RV) dilation and dysfunction secondary to chronic severe pulmonary regurgitation are well documented in patients late after repair of tetralogy of Fallot (TOF)^1–3^. On the other hand, increasing evidence suggests that left ventricular (LV) dysfunction may also have prognostic significance in these patients^4^. Left ventricular systolic dysfunction has been attributed to several factors including preoperative hypoxaemia^5^, LV fibrosis^6, 7^, LV dyssynchrony^8^ and adverse right-left ventricular interaction^9^. On the other hand, the phenotype and understanding of pathogenetic mechanism of LV diastolic dysfunction in repaired TOF is less clear.

Diastolic ventricular dysfunction may be related to relaxation abnormality and/or stiffening of the myocardium. Previous studies in repaired TOF patients have primarily assessed indirectly LV early diastolic relaxation and estimated late diastolic filling using Doppler imaging^10, 11^. Importantly, however, recent data suggest that potential alteration of myocardial substrates occurs in repaired TOF, which may predispose to stiffening of LV myocardium. In these patients, increased levels of circulating biomarkers of collagen synthesis have been reported^12, 13^. Cardiac magnetic resonance (CMR) imaging with late gadolinium enhancement^6^ and T1 mapping^7^ techniques has further revealed evidence of LV fibrosis. The potential consequence on LV stiffness of LV remodeling related to myocardial fibrosis and geometric eccentricity secondary to RV volume overload has, however, not been explored in patients after TOF repair.

Recently, there has been increasing use of echocardiography for non-invasive assessment of myocardial stiffness. The parameter of diastolic wall strain (DWS)^14^, which quantifies the thinning of myocardium during diastole, has been found to be useful in assessing diastolic myocardial stiffness and prognostication in heart failure patients with preserved ejection fraction^15^. Another potentially useful LV stiffness index that relates Doppler-estimated LV filling pressure to LV end-diastolic dimension has been used to interrogate myocardial stiffness in athletes^16^. In this study we aimed to utilize these indices to test the hypothesis that LV stiffness is altered in adolescents and young adults with repaired TOF and to determine its relationship to myocardial calibrated integrated backscatter (cIB) as a marker of fibrosis^17^ and LV diastolic myocardial deformation.

## Methods

### Subjects

This was a retrospective study of our echocardiographic database of congenital heart disease that included 77 consecutively studied TOF patients post total surgical repair. The following patient data were retrieved from case notes: cardiac and associated lesions, type of operation performed, date and age of operation, and the need for additional procedures. A total of 80 age-matched healthy controls were retrieved from the healthy control database for the purpose of comparisons. These included healthy staff volunteers, their friends, and subjects with chest pain or palpitation for which no organic causes had been identified. The body weight and height of all subjects were measured at the time of echocardiographic acquisitions and the body surface area was calculated accordingly. The total number of around 80 subjects in each group gave a statistical power of 80% with 5% level of significance with two-sided testing to detect a minimum DWS difference of 0.03 with a variation of 0.08 based on the previously reported standard deviation^15^. This study was approved by the Institutional Review Board of the University of Hong Kong/Hospital Authority Hong Kong West Cluster, Hong Kong, and all of the methods as described were performed in accordance with the approved guidelines and regulations. Informed consent had been obtained from all of the participants at the time of acquisition of echocardiographic images.

### Conventional echocardiographic assessment

Echocardiographic acquisitions were made using Vivid 7 ultrasound machine (General Electric, Vingmed, Horten, Norway). Offline analyses of the recordings were performed using EchoPAC software (General Electric, Vingmed, Horten, Norway). Measurements of all echocardiographic parameters were made in three cardiac cycles and the average was taken for statistical analyses.

From the four-chamber view, RV end-diastolic and end-systolic areas were measured and RV fractional area change was calculated accordingly. Transmitral pulsed-wave Doppler examination was performed to obtain peak early diastolic inflow velocity (E), late diastolic inflow velocity (A), E/A ratio, and E deceleration time. Tissue Doppler echocardiography was performed with sample volume positioned at the basal LV free wall-mitral annular junction to obtain the peak early diastolic myocardial tissue velocity (*e*), late diastolic myocardial tissue velocity (*a*), *e*/*a* ratio, and E/*e* ratio. Severity of pulmonary regurgitation was graded semi-quantitatively as mild, moderate, or severe by color flow mapping^18^.

### Assessment of LV stiffness

Based on M-mode assessment of the parasternal short-axis view, the LV posterior wall (LVPW) thickness at systole and diastole was determined. Diastolic wall strain (DWS) was calculated as (LVPW at systole - LVPW at diastole)/LVPW at systole^14^. This parameter reflected thinning of the LV posterior wall during diastole. Based on the linear elastic theory, this change in wall thickness reflected resistance to deformation in diastole and hence myocardial stiffness^14^. Our group had previously reported on high reproducibility of DWS measurements^19^.

Stiffness index was calculated as (E/*e*)/LVEDd, where LVEDd is LV end-diastolic dimension derived from M-mode measurement^16^. This parameter was used to provide an estimate of the pressure-to-volume relationship, with LV filling measure being estimated by E/*e* ratio and end-diastolic LV volume being estimated by LVEDd.

### Measurement of cIB

Integrated backscatter of the ventricular septum and posterior LV wall was measured from the parasternal short-axis view at the papillary muscle level at end-diastole as described previously^20^. The sample volume was tracked manually to maintain the same region throughout the cardiac cycle. Calibrated integrated backscatter was calculated as the difference between integrated backscatter at the two regions and that at the pericardium. The average cIB at the two sites was taken for statistical analysis. Our group has previously reported on high reproducibility of cIB measurement^20^.

### Quantification of LV diastolic myocardial deformation

Left ventricular diastolic myocardial deformation in the longitudinal dimension was interrogated using speckle tracking echocardiography as reported previously^21^. By tracking the entire LV contour, the global LV longitudinal early and late diastolic strain rates were determined from the apical four chamber view using EchoPAC software (GE Medical Systems).

### Statistical analysis

Data was expressed as mean ± standard deviation. Differences in demographic and echocardiographic parameters between repaired TOF patients and controls were compared using the Student’s t-test and Fisher’s exact test where appropriate. Relationships between LV stiffness indices and cIB and indices of LV diastolic deformation were explored using Pearson correlation analysis. Multivariate analysis using multiple linear regression was performed to determine significant correlates of DWS and stiffness index respectively with adjustments of variables having a p value < 0.05 by univariate analysis. A p value < 0.05 was considered statistically significant. All statistical analyses were performed using SPSS version 17.0 (SPSS, Inc., Chicago, IL, USA).

## Results

### Subjects

Of the 77 patients, 72 had TOF with pulmonary stenosis, while 5 had pulmonary atresia. The patients (43 males) were aged 18.5 ± 8.1 years and studied at 14.6 ± 7.3 years after total surgical repair. Twenty patients had systemic-to-pulmonary arterial shunt palliation before total surgical repair. Of the 72 TOF patients with pulmonary stenosis, 58 required transannular patch repair of RV outflow. Pulmonary valve replacement was performed subsequently in four of the 77 patients. Six of the patients had syndromal associations including DiGeorge syndrome in 3, Down syndrome in 1, trisomy 20p in 1, and VATER association in 1. The 80 controls (41 males) were aged 16.5 ± 7.0 years (p = 0.11). Compared with controls, patients had similar body weight (46.2 ± 16.3 kg vs 49.9 ± 16.3 kg, p = 0.18), body height (152.0 ± 17.7 cm vs 155.0 ± 22.0 cm, p = 0.35), and body surface area (1.38 ± 0.33 m^2^ vs 1.46 ± 0.34 m^2^, p = 0.14).

### Conventional and speckle tracking echocardiographic findings

Table 1 summarized the echocardiographic findings in patients and controls. The RV end-diastolic area indexed by body surface area was significantly greater in patients than controls (p < 0.001), although there was no significant difference in RV fractional area change between the two groups (p = 0.14). Of 77 repaired TOF patients, 11 (14.3%), 14 (18.2%) and 52 (67.5%) had respectively mild, moderate and severe degree of pulmonary regurgitation.

**Table 1:** Echocardiographic indices in patients and controls.

	Patients (n = 77)	Controls (n = 80)	p
*Mitral inflow Doppler indices*			
E (cm/s)	97.6 ± 21.7	97.8 ± 22.5	0.94
A (cm/s)	44.7 ± 14.8	50.8 ± 14.3	0.01*
E/A ratio	2.37 ± 0.85	2.02 ± 0.51	0.002*
E deceleration time (ms)	155 ± 52	147 ± 34	0.28
*Mitral annular tissue Doppler*			
*e* (cm/s)	15.0 ± 3.71	17.5 ± 2.5	<0.001*
*a* (cm/s)	6.0 ± 2.0	6.6 ± 1.5	0.02*
*e*/*a* ratio	2.70 ± 1.03	2.75 ± 0.72	0.71
E/*e* ratio	6.95 ± 2.41	5.66 ± 1.30	<0.001*
*LV longitudinal diastolic deformation*			
SRe (/s)	1.52 ± 0.45	2.05 ± 0.52	<0.001*
SRa (/s)	0.59 ± 0.19	0.72 ± 0.19	<0.001*
*2D echocardiographic measurements*			
RV EDA (cm^2^/m^2^)	17.6 ± 4.7	9.7 ± 2.1	<0.001*
RV FAC (%)	54.4 ± 9.1	56.4 ± 7.0	0.14
LVEDd (mm)	39.8 ± 7.8	43.2 ± 5.4	0.002*

A, transmitral late diastolic inflow velocity; a, late diastolic annular myocardial velocity; E, transmitral early diastolic inflow velocity; e, early diastolic annular myocardial velocity; FAC, fractional area change; LV, left ventricular; LVEDd, left ventricular end-diastolic dimension; RV, right ventricular; SRa, left ventricular late diastolic strain rate; RV EDA, RV end diastolic area; SRe, left ventricular early diastolic strain rate. *Statistically significant.

Worse LV diastolic mechanics in repaired TOF patients was evidenced by the significantly lower *e* velocity (p < 0.001) and global longitudinal early (p < 0.001) and late (p < 0.001) diastolic strain rates, and greater E/*e* ratio (p < 0.001) in patients as compared with those of controls.

### LV stiffness and cIB

**Figure 1:**
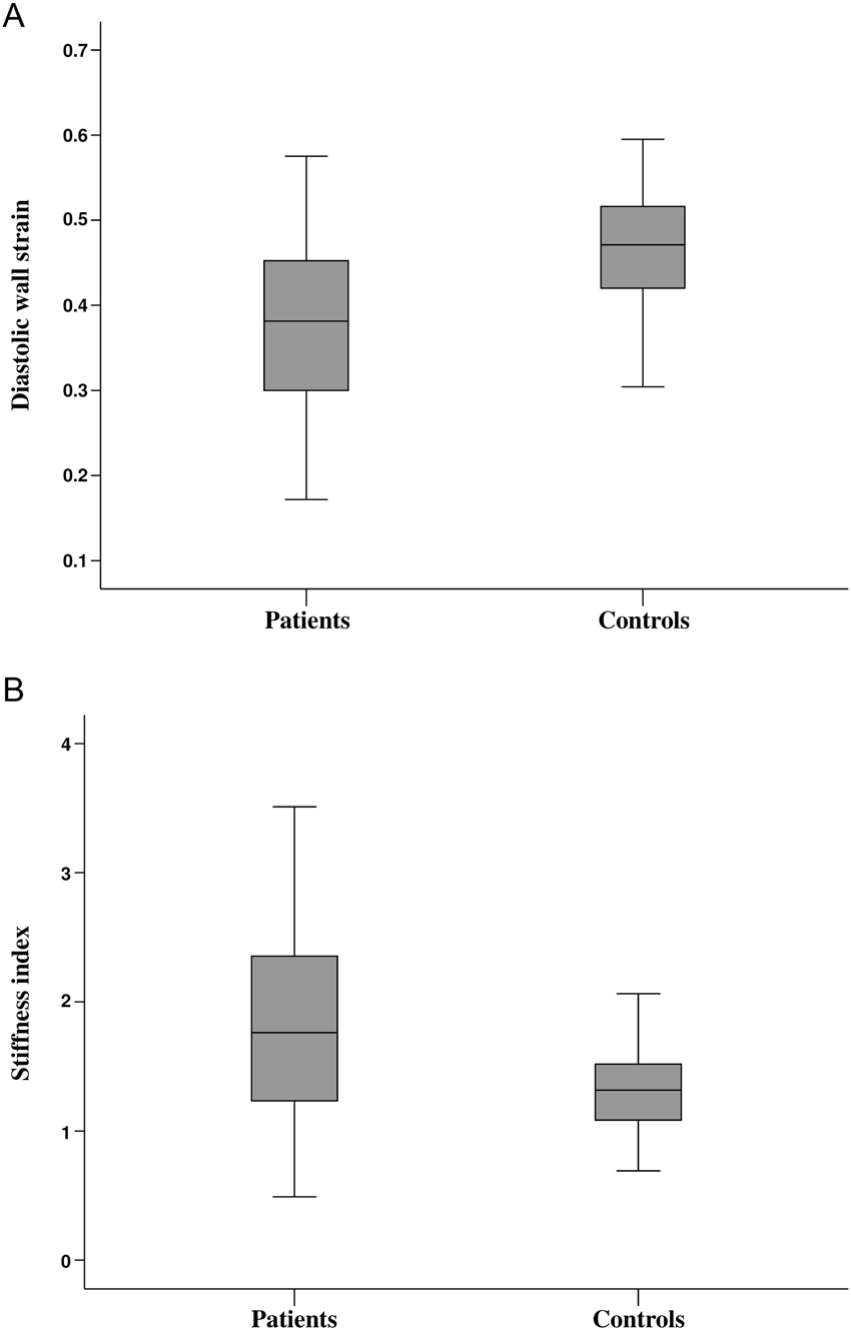
Boxplots showing (**A)** diastolic wall strain and (**B**) stiffness index of the left ventricle in patients and controls.

Compared with controls, TOF patients had significantly lower DWS (0.38 ± 0.10 vs 0.47 ± 0.08, p < 0.001) and higher LV stiffness index (1.82 ± 0.71 vs 1.34 ± 0.38, p < 0.001) (Fig. 1A,B). For the entire cohort, DWS was found to correlate negatively with LV stiffness index (r = −0.31, p < 0.001).

For cIB, patients had significantly greater septal cIB (−19.4 ± 6.6 dB vs −24.9 ± 5.3 dB, p < 0.001), LV posterior wall cIB (−19.4 ± 4.8 dB vs −21.3 ± 4.6 dB, p = 0.01) and average cIB (−19.4 ± 4.7 dB vs −23.1 ± 4.2 dB, p < 0.001).

The septal but not posterior wall cIB was found to correlate negatively with LV DWS (r = −0.21, p = 0.01). Additionally, cIB at septum was found to correlate with mitral annular *e* velocity (r = −0.17, p = 0.03), LV early diastolic longitudinal strain rate (r = −0.29, p < 0.001), RV volume load (r = 0.24, p = 0.002), and RV fractional area change (r = −0.21, p = 0.007). These were, however, no significant correlation between septal or posterior cIB and LV stiffness index.

### Correlates of LV stiffness

**Figure 2:**
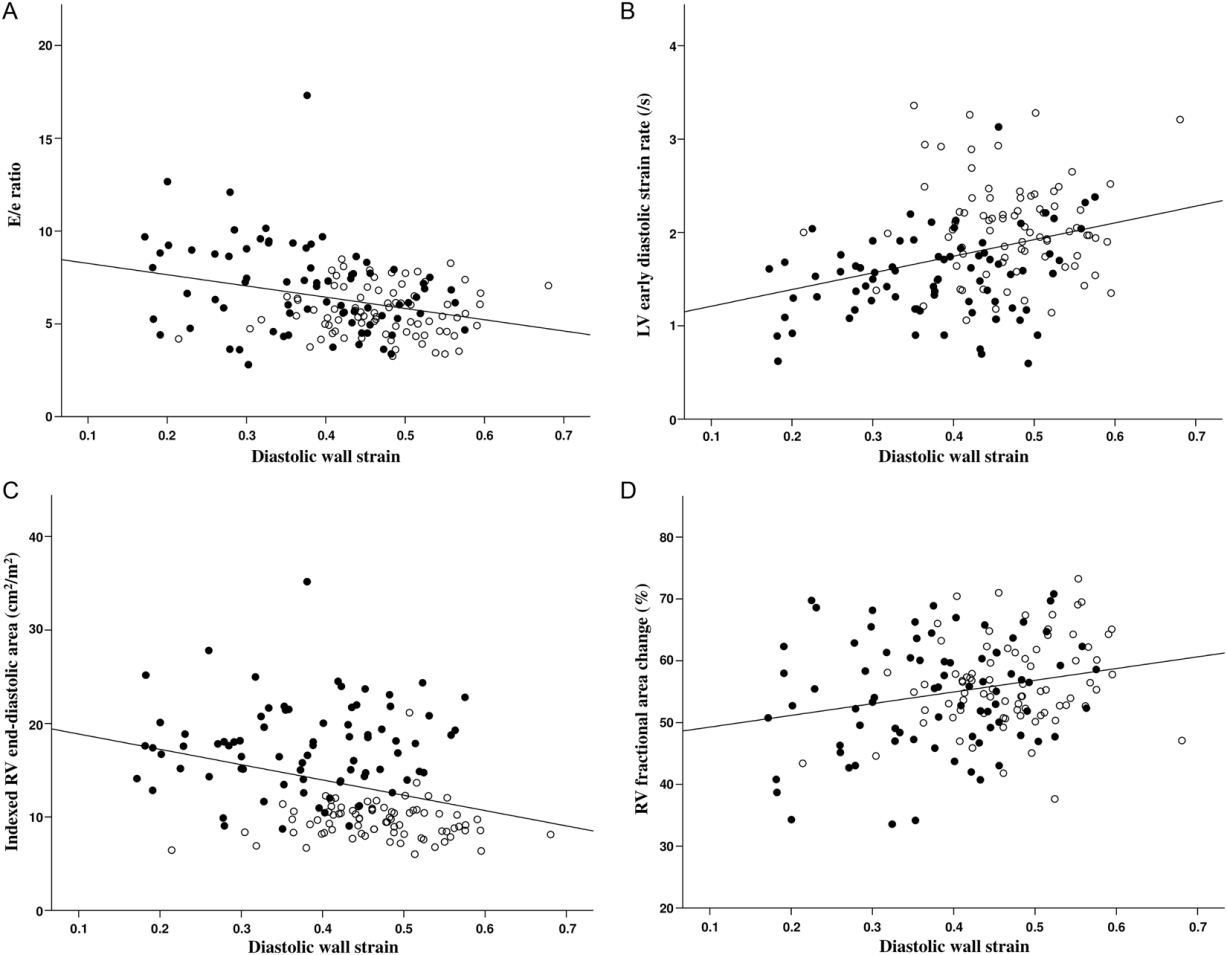
Scatterplots showing the relationships between left ventricular diastolic wall strain and (**A**) the ratio of transmitral early diastolic flow velocity (**E**) to mitral annular early diastolic velocity (e), (**B**) left ventricular (LV) early diastolic strain rate, (**C**) indexed right ventricular (RV) end-diastolic area, and (**D**) RV fractional area change. Solid circles represent patients, empty circles represent controls.

Table 2 summarized the correlation analyses of LV DWS and stiffness index. Left ventricular DWS was found to correlate negatively with E/*e* ratio (p < 0.001) and RV end-diastolic area (p < 0.001), and positively with *e* velocity (p < 0.001), LV global early (p < 0.001) and late (p = 0.01) diastolic longitudinal strain rates, and RV fractional area change (p = 0.003) (Fig. 2A–D). Multivariate analysis identified E/*e* ratio (p = 0.008), RV end-diastolic dimension (p = 0.02), and RV fractional area change (p = 0.01) as significant independent correlates of DWS.

**Table 2:** Correlation between left ventricular stiffness and indices of left and right ventricular function.

	Diastolic wall strain				Stiffness index			
	*Univariate correlation*		*Multiple regression*		*Univariate correlation*		*Multiple regression*	
	r	p	beta	p	r	p	beta	p
*Mitral inflow Doppler indices*								
E	−0.04	0.66			0.55	<0.001*	0.02	0.57
A	0.07	0.36			0.13	0.10		
E/A ratio	−0.08	0.32			0.34	<0.001*	−0.01	0.57
E deceleration time	−0.05	0.56			0.09	0.28		
*Mitral annular tissue Doppler*								
* e*	0.31	<0.001*	−0.03	0.78	−0.60	<0.001*	0.03	0.52
* a*	0.16	0.045*	0.05	0.54	−0.30	<0.001*	−0.04	0.26
* e*/*a*	0.10	0.22			−0.19	0.02*	−0.05	0.15
E/*e* ratio	−0.30	<0.001*	−0.26	0.008*	0.90	<0.001*	0.80	<0.001*
*LV longitudinal diastolic deformation*								
SRe	0.33	<0.001*	0.16	0.11	0.02	0.83		
SRa	0.20	0.01*	0.01	0.90	−0.13	0.11		
*2D echocardiographic measurements*								
Indexed RV EDA	−0.31	<0.001*	−0.20	0.02*	0.32	<0.001*	0.43	0.004*
RV FAC	0.24	0.003*	0.18	0.01*	0.01	0.88		
LVEDd	0.11	0.16			−0.64	<0.001*	−0.40	<0.001*

Abbreviation as in Table 1. *Statistically significant.

On the other hand, the stiffness index expectedly was found to correlate with LV end-diastolic dimension (p < 0.001), transmitral E velocity (p < 0.001), mitral annular *e* velocity (p < 0.001), and E/*e* ratio (p < 0.001) based on which the parameter was derived. Additionally, stiffness index was found to correlate positively with RV end-diastolic area (r = 0.32, p < 0.001). Multivariate analysis identified RV end-diastolic area (p = 0.004), LV end-diastolic dimension (p < 0.001) and E/*e* ratio (p < 0.001) as significant independent correlates.

In patients, there were no significant correlations between severity of pulmonary regurgitation and DWS and stiffness index (both p > 0.05).

## Discussion

The present study demonstrates diastolic myocardial stiffening as reflected by reduced LV diastolic wall strain and increased stiffness index in adolescents and young adults with repaired TOF. Increased myocardial stiffness was associated with worse LV diastolic myocardial deformation as characterized by slower global LV early and late diastolic longitudinal strain rates. Furthermore, our data provided evidence that myocardial fibrosis and RV volume overload might be important factors that contributed to LV stiffening late after repair of TOF. To our knowledge, this is the first study to explore LV stiffening and its possible associations with myocardial fibrosis, RV volume load, and LV diastolic myocardial deformation.

There has been increasing interest on ventricular diastolic dysfunction in patients with repaired TOF. Previous studies have focused on interrogation of LV relaxation abnormalities^10, 11^. Our findings of reduced mitral annular *e* velocity and global LV longitudinal diastolic strain rate are compatible with impaired LV relaxation and agree with those reported previously^11, 12^. Increased LV end-diastolic pressure as documented by cardiac catheterization in repaired TOF patients, on the other hand, provides evidence of reduced LV compliance^22^, although data on stiffening of ventricular myocardium in these patients are lacking.

Direct assessment of LV stiffness is challenging and requires invasive cardiac catheterization for the derivation of the diastolic pressure-volume curve and calculation of chamber stiffness constant^23^. Recently, the echocardiographic parameter of DWS, which reflects the change of wall thickness and resistance to deformation during diastole, has been validated in animal studies and shown to correlate inversely with the invasively determined myocardial stiffness constant^14^. In clinical studies, DWS has been shown to predict outcomes of patient with heart failure with preserved^15^ and reduced^24^ ejection fraction and in patients with non-ST elevation myocardial infarction^25^. With regard to the LV stiffness index, which provides an estimate of the relationship between LV filling pressure and end-diastolic volume, this has been reported to be useful in the demonstration of reduced myocardial stiffness in elite athletes^16^. Our findings of reduced DWS and concomitant increased LV stiffness index in patients with repaired TOF provide therefore evidence of LV myocardial stiffening. Importantly, the magnitude of LV myocardial stiffening was found to relate inversely to indices of LV diastolic deformation. Hence, LV diastolic dysfunction in patients with repaired TOF is attributable in part to increased stiffness of the ventricular myocardium.

While the exact mechanisms of LV myocardial stiffening in our patients remain to be unveiled, the present study suggests that two factors may potentially be important. Firstly, increased cIB of the ventricular septum and LV posterior in our patients suggests that alteration of myocardial substrate with increased deposition of fibrous tissue might be a culprit. Calibrated integrated backscatter has been used to assess myocardial fibrosis in obese subjects^26^, in patients with metabolic syndrome^27^, and in those with heart failure^28^. Our finding of increased cIB in repaired TOF patients are compatible with previous reports of increased levels of circulating biomarkers of collagen synthesis^12, 13^ and CMR late gadolinium enhancement^6^ and T1 mapping^7^ studies demonstrating evidence of LV fibrosis in these patients. Fibrosis increases the viscoelastic burden of the myocardium and impairs relaxation, diastolic suction, and passive stiffness^29^. Indeed, significant associations between myocardial fibrosis and worsening of LV stiffness have been shown in heart transplant recipients^30^ and in patients with heart failure with preserved ejection fraction^31^.

Intriguingly, we found significant negative correlation between DWS and cIB of the septum but not posterior wall. Furthermore, the septal cIB difference between patients and controls was more significant than that of the LV posterior cIB difference. It is interesting to note that preferential fibrotic alteration of the septum has also been shown in patients with metabolic syndrome and LV dysfunction^27^ and in patients with heart failure with preserved ejection fraction^28^. In our patients, previous cardiopulmonary bypass and surgical closure of ventricular septal defect might have accounted for our findings although this remains speculative. Notwithstanding, preferential involvement of the septum has been shown to influence adversely the degree of LV diastolic dysfunction^32^.

The second potentially important factor that may contribute to LV stiffness in our patients is geometric alteration of the ventricles secondary to RV volume overload. Multivariate analysis has identified RV end-diastolic area and fractional area change as independent determinants of both DWS and stiffness index. Our hypothesis is further supported by the study of Schwartz *et al*. who found a significant relationship between RV end-diastolic volume and LV end-diastolic pressure^22^. Kim *et al*. have recently further reported using CMR LGE that non-ischaemic septal fibrosis is independently associated with RV chamber dilation and hypothesized the role of increased RV wall stress^33^. Additionally, reduced LV preload secondary to altered septal geometry may reduce ventricular compliance as reported similarly in patients with large atrial septal defects^34^.

While the prognostic implication of LV systolic dysfunction in repair TOF has been documented^4^, the clinical implication of LV diastolic dysfunction in repaired TOF is less clear. Nonetheless, several considerations warrant comments. Left ventricular dysfunction may impact adversely on pulmonary arterial compliance^35^, which in turn increases RV afterload and potentially worsens pulmonary regurgitation^36^. This is of particular relevance in the context of repaired TOF. Utilization of a semi-quantitative method rather than cardiac magnetic resonance to quantify pulmonary regurgitation in this study, however, might have precluded the identification of an association between LV myocardial stiffness and pulmonary regurgitation. Increased LV end-diastolic pressure of 12 mmHg or more has been associated with larger right ventricles^22^ and shown to be strong predictor of appropriate implantable cardioverter-defibrillator shocks in patients with repaired TOF^37^. The inclusion of LV diastolic dysfunction when considering the timing of pulmonary valve replacement and assessment of it reversibility after valve replacement should be topics for further research.

Several limitations to the study require comments. Firstly, this cross-sectional study cannot provide data on the relationship between progressive RV dilation and LV stiffening. Further longitudinal studies are required to address the serial changes in LV stiffness post TOF repair and its potential improvement after pulmonary valve replacement. Secondly, the measurement of DWS is regional, based on changes of the LV posterior wall thickness in the cardiac cycle. Heterogeneity of biomechanical properties in different myocardial regions may exist and further work to develop a more global echocardiographic index of myocardial stiffness is indicated. We have therefore also included the LV stiffness index, albeit based the estimates of LV filling pressure and size, to provide a more global assessment. It would have been ideal to be able to assess RV stiffness using similar echocardiographic measurements. However, the use of DWS to quantify RV stiffness has not been reported and yet to be validated. Finally, the use of the new CMR T1 mapping technique, which was not available at the time of study of our patients, may shed more light on the relationship between diffuse myocardial fibrosis and LV diastolic mechanics.

In conclusion, LV stiffening occurs late after repair TOF repair and is related to impaired LV diastolic myocardial deformation, myocardial calibrated integrated backscatter, and RV volume overload.
